# Oncostatin M exerts a protective effect against excessive scarring by counteracting the inductive effect of TGFβ1 on fibrosis markers

**DOI:** 10.1038/s41598-019-38572-0

**Published:** 2019-02-14

**Authors:** Vincent Huguier, Jean-Philippe Giot, Marie Simonneau, Pierre Levillain, Sandrine Charreau, Martine Garcia, Jean-François Jégou, Charles Bodet, Franck Morel, Jean-Claude Lecron, Laure Favot

**Affiliations:** 1 Laboratoire Inflammation, Tissus Epithéliaux et Cytokines, EA4331, Université de Poitiers, 86073 POITIERS, France; 2 Centre Hospitalier Universitaire de Poitiers, 86021 Poitiers, France; 3 Chirurgie Plastique et Maxillo-faciale, Centre Hospitalier Universitaire de Grenoble, Hôpital Michallon, 38700 La Tronche, France

## Abstract

Wound healing is a complex physiological process that repairs a skin lesion and produces fibrous tissue. In some cases, this process can lead to hypertrophic scars (HS) or keloid scars (KS), for which the pathophysiology remains poorly understood. Previous studies have reported the presence of oncostatin M (OSM) during the wound healing process; however, the role of OSM in pathological scarring remains to be precisely elucidated. This study aims to analyse the presence and involvement of OSM in the pathological scarring process. It was conducted with 18 patients, including 9 patients with hypertrophic scarring and 9 patients with keloid scarring. Histological tissue analysis of HS and KS showed minor differences in the organization of the extracellular matrix, the inflammatory infiltrate and the keratinocyte phenotype. Transcriptomic analysis showed increased expression levels of fibronectin, collagen I, TGFβ1, β-defensin-2 and S100A7 in both pathological samples. OSM expression levels were greater in HS than in KS and control skin. *In vitro*, OSM inhibited TGFβ1-induced secretion of components of the extracellular matrix by normal and pathological fibroblasts. Overall, we suggest that OSM is involved in pathological wound healing processes by inhibiting the evolution of HS towards KS by controlling the fibrotic effect of TGFβ1.

## Introduction

Skin has multiple functions, such as acting as a barrier for pathogens, regulating body temperature and preventing dehydration. In this respect, while cutaneous wound healing is a critical survival factor, the condition of the resulting scar is not vital but remains essential for aesthetic appearances and self-esteem. Hypertrophic scars (HS) and keloid scars (KS) can alter cutaneous mechanical characteristics and lead to functional anomalies.

Normal wound healing is a tightly regulated inflammatory process involving cellular and molecular interactions between immune cells, keratinocytes, fibroblasts, extracellular matrix components and soluble mediators coordinating fibroplasia, epithelialization and angiogenesis^1^. Pathologic responses leading to fibrosis or chronic ulcers may occur if any part of the healing process is altered. HS and KS both result from abnormal wound healing processes in which tissue repair- and regeneration-regulating mechanisms are altered or deregulated.

HS are characterized by raised fibrous lesions that spontaneously regress, while KS are the result of an overgrowth of fibrous tissues without spontaneous regression of the process. In addition, tissue extends beyond the borders of the original wound in KS^2^.

After cutaneous injury, a cascade of events, including overlapping phases (inflammation, cell proliferation, extracellular matrix deposition and remodelling), leads to physiological tissue repair. This wound healing process is initiated and tightly coordinated by specific cytokines, chemokines and growth factors mainly secreted by keratinocytes, fibroblasts and resident or recruited immune cells^3^. The molecular mechanisms involved in HS and KS are not fully understood. Several studies have suggested that excessive scarring is the result of a prolonged proliferative phase and delayed remodelling phase^4^ and that dermal fibroblasts substantially contribute to the process of fibrosis^5^.

In fibrosis induction, the ability of TGFβ1 to stimulate collagen synthesis appears to be central and plays important roles in extracellular matrix (ECM) accumulation during abnormal wound healing^1,6^. Nonetheless, other cytokines, such as IL-1, IL-4, IL-6 and oncostatin M (OSM), also control this process. IL-4, which is mainly produced by Th2 lymphocytes, directly stimulates collagen and fibronectin production by fibroblasts, and increased IL-4 levels have been reported in HS^7^. IL-1α and IL-1β, which are mainly produced by macrophages but also by keratinocytes and fibroblasts, are known to induce extracellular matrix degradation and decrease collagen production. IL-1α and IL-1β can target both keratinocytes and fibroblasts, modulating cell proliferation^8,9^. IL-6, which is an important pleiotropic proinflammatory cytokine targeting both keratinocytes and fibroblasts, is enhanced in KS^10,11^. IL-6 inhibits matrix metalloproteinase expression and induces fibronectin and collagen synthesis by fibroblasts^10^. OSM belongs to the cytokines of the IL-6 family and is a pleiotropic cytokine secreted by T cells, monocytes/macrophages, dendritic cells and neutrophils^12^. OSM has been implicated in fibrotic diseases in the lung, liver, heart, vessels, kidney, pancreas and skin^12–16^ by modulating the fibroblastic phenotype and ECM protein production^14^. During skin repair, OSM expression is induced in the early inflammatory phase^17^. In diabetic ob/ob mice, hyperexpression of OSM in wounded skin is associated with impaired healing conditions^17^.

In human skin pathological situations, such as psoriasis, atopic dermatitis and hypertensive leg ulcers (HLUs), OSM is overexpressed^18–20^. OSM exerts proinflammatory activities, stimulates keratinocyte migration and increases the thickness of reconstituted epidermis *in vitro*^18,20–23^. *In vivo*, the intradermal injection of OSM encoding adenoviruses in the mouse ear has been shown to induce substantial skin inflammation with epidermal thickening associated with increased basal keratinocyte proliferation^22^.

Considering the properties of this cytokine, we investigated the putative role of OSM in the physiopathology of hypertrophic and keloid scarring. We compared pathological hypertrophic and keloid scar tissues to normal skin in terms of histology, skin inflammation markers and extracellular matrix mRNA and protein expression. The ability of OSM to modulate the expression of ECM proteins involved in skin fibrosis was further investigated *in vitro* on primary dermal fibroblasts.

## Results

### Characteristics of the patients

Eighteen patients presenting pathologic scars were included in the study (Table 1). None of the 9 patients presenting typical HS had previous scar treatment. These scars were secondary to a previous surgery with a mean delay of 7.9 months. Two patients had general medication for diabetes, high blood pressure or dyslipidaemia. The sex ratio (male/female) was 0.5, and the mean age was 35.3 years. Six of the 9 patients presenting typical KS had a previous injection of a corticosteroid into the scar more than 2 years before surgery and sampling. All KS were active when the biopsies were performed. One patient was treated with levothyroxine, and another patient was treated with insulin. The KS were secondary to a previous trauma or surgery and were resected after a median delay of 69 months. The biopsies were collected from the central part of the scar, and the entire thickness of the scar was collected. The male/female sex ratio was 0.8, and the mean age was 29.7 years.

**Table 1:** Clinical data of hypertrophic scar and Keloid scar patients.

Patient	Scar type	Age	Sex	Associated pathologies	General treatment	Scar localisation	Scar age
1	HS	73	M	type II diabetes HTA dyslipidemia	Glitazid Furosemid Hydrochlorothiazid	Face	3 months
2	HS	19	F	Asthma	None	Arm	3 months
3	HS	18	F	None	None	Abdomen	11 months
4	HS	40	M	None	None	Abdomen	5 months
5	HS	18	F	None	None	Arm	9 months
6	HS	18	F	None	None	Forearm	11 months
7	HS	50	M	None	None	Face	11 months
8	HS	48	F	None	None	Abdomen	6 months
9	HS	34	F	None	None	Abdomen	12 months
10	KS	30	F	None	None	Abdomen	2 years
11	KS	39	F	Hypothyroidism	Levothyroxin	Abdomen	2 years
12	KS	47	M	None	None	Face	15 years
13	KS	20	M	Type I diabetes	Insulin	Forearm	1.5 years
14	KS	30	M	None	None	Anterior chest	10 years
15	KS	25	F	None	None	Ear	1.5 years
16	KS	28	M	None	None	Ear	8 years
17	KS	20	F	None	None	Ear	8 years
18	KS	29	F	None	None	Abdomen	5 years

HS: Hypertrophic scar. KS: Keloid scar.

### Histopathology of the scars

**Figure 1:**
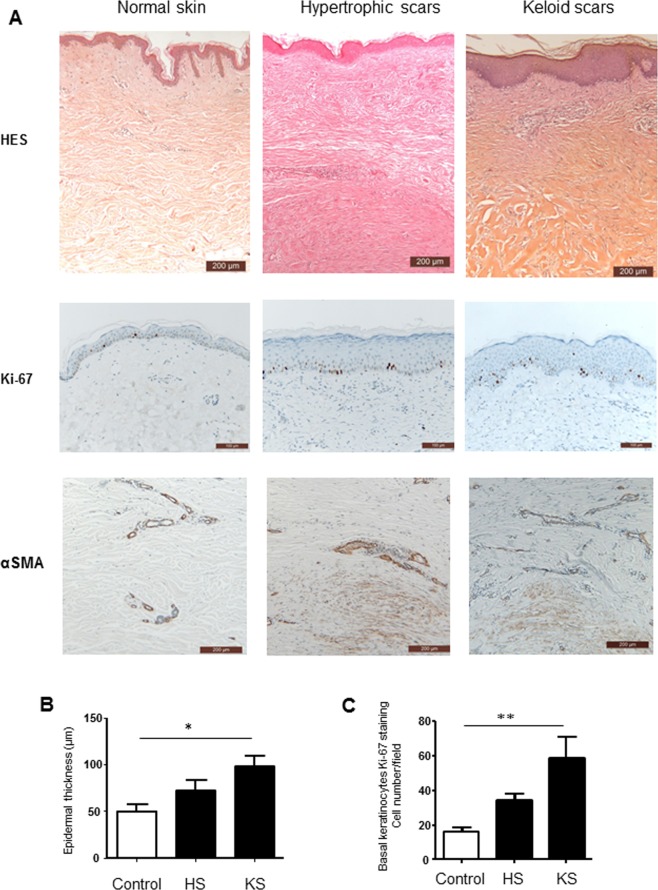
Histological analysis of pathologic scars. (**A**) Skin biopsies from normal skin, hypertrophic scars and keloid scars were fixed and embedded in paraffin, and 4 µm sections were stained with haematoxylin and eosin, Safran (HES), Ki-67 or αSMA. Scale bars indicate 200 µm for HES and αSMA staining and 100 µm for Ki-67 staining. (**B**) Epidermal thickness is presented as the mean of three measurements per subject (n = 5). C: The number of basal keratinocytes expressing Ki-67 antigen (n = 5), ^*^p < 0.05; ^**^p < 0.01.

Control healthy skin was characterized by a thin epidermis (49.86 +/− 7.6 µm) with typical rete pegs (Fig. 1). In both types of pathologic scars, the epidermis appeared thicker (72.60 +/− 10.9 µM in HS and 98.26 +/− 11.51 µm in KS), with a flattening of the dermo-epithelial junction (Fig. 1A,B). We observed thin collagen bundles in the papillary dermis that were aligned parallel to the epithelial surface. In both types of scars, the mid and reticular dermis were fibrous with thick collagen bundles and were hyalinized in the keloid scars. Infiltrated immune cells in the dermis consisted of lymphocytes, eosinophils, mastocytes and macrophages, with no quantitative or qualitative differences between the two types of scars (Table 2). In normal skin epidermis samples, a small proportion of basal keratinocytes were observed to be proliferating (16 cells +/− 2.6 per microscopic field), as indicated by Ki67 staining. The number of proliferative keratinocytes was greater in HS (34.20 cells +/− 3.83 per microscopic field) and KS (59.17 cells +/− 11.7 per microscopic field) and significantly greater in KS (p < 0.01, Fig. 1C).

**Table 2:** Histologic quantification of immune infiltrate and immunohistochemical quantification of αSMA scored as follow: 0 not expressed, +weekly expressed, ++strongly expressed, +++very strongly expressed.

	αSMA	Hyalinized collagen fibers	Lymphocytes	Neutrophils	Eosinophils-mastocytes	Macrophages	Fibroblastes
**Keloid scars**							
**a**	**+**	**+++**	**+**	**0**	**+**	**+**	**+**
**b**	**+**	**++**	**++**	**0**	**0**	**+**	**++**
**c**	**+**	**+++**	**+++**	**0**	**+**	**+**	**+**
**d**	**+**	**+++**	**+**	**0**	**0**	**0**	**+**
**e**	**0**	**+++**	**+**	**0**	**+**	**0**	**+**
**Hypertrophic scars**							
**a**	**+++**	**+**	**0**	**0**	**0**	**0**	**++**
**b**	**+++**	**+**	**0**	**0**	**0**	**0**	**++**
**c**	**0**	**0**	**+**	**0**	**0**	**0**	**+**
**d**	**+**	**++**	**+**	**0**	**0**	**+**	**+**
**e**	**++**	**0**	**++**	**0**	**+**	**+**	**++**

In both types of scars, we observed an αSMA expression in myofibroblasts of the mid and reticular dermis, albeit more numerous in HS (Fig. 1A, Table 2).

### OSM was overexpressed in hypertrophic scars but not in keloid scars

Immune cells, fibroblasts and keratinocytes secrete many cytokines, growth factors and chemokines that are critical for the wound healing process. We quantified the expression of factors that have been shown to be active on keratinocytes and fibroblasts by qRT-PCR.

**Figure 2:**
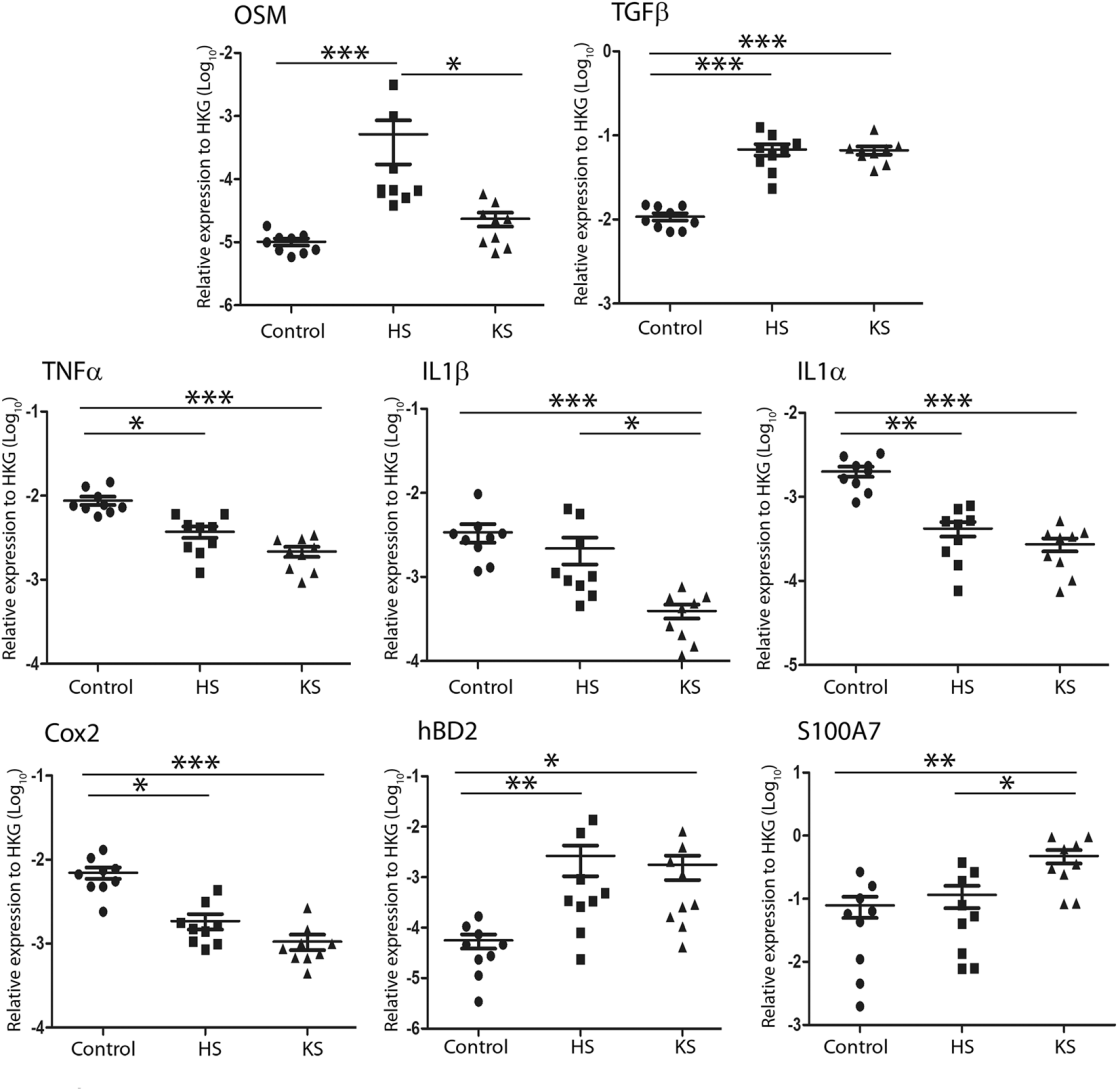
OSM was overexpressed in hypertrophic but not in keloid scars. Cytokine and antimicrobial peptide mRNA relative expression in normal skin (control), hypertrophic scars (HS) and keloid scars (KS) (n = 9) was analysed by qRT-PCR using GAPDH and β-actin as housekeeping genes to normalize gene expression. The y-axis represents the relative expression normalized to housekeeping genes (HKGs). ^*^p < 0.05; ^**^p < 0.01; ^***^p < 0.001.

As indicated in Fig. 2, OSM expression was specifically upregulated in HS (50-fold greater compared to the control, p < 0.001) but not in KS, while TGFβ1 expression levels were elevated in both pathologic scars (6-fold increase compared to the control, p < 0.001). IL-6, IL-17A and IL-22 were not detected in normal skin or in HS or KS (data not shown). The expression of TNFα, IL-1α and Cox2 mRNA was downregulated in KS and in HS compared to control skin. IL-1β mRNA expression levels were similar in control skin and in HS, while it was significantly lower in KS (9-fold decrease, p < 0.001)

We also analysed the expression of the inflammatory molecules S100A7 and BD2, which are known to be upregulated by inflammatory cytokines in skin^24^. S100A7 and BD2 were upregulated in both types of scars.

### ECM protein expression was upregulated in hypertrophic and keloid scars

**Figure 3:**
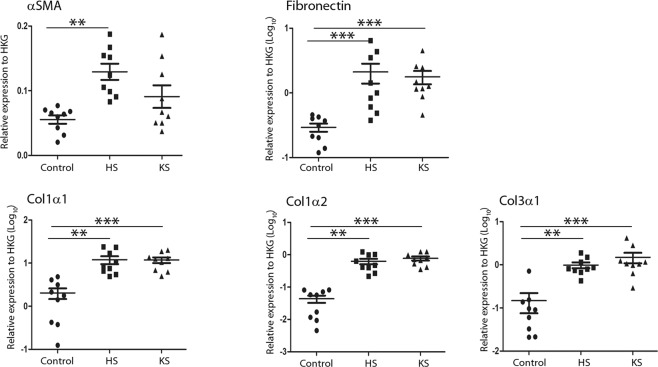
ECM protein expression was upregulated in hypertrophic and keloid scars. Extracellular matrix protein mRNA relative expression in normal skin (control), hypertrophic scars (HS) and keloid scars (KS) (n = 9) was analysed by qRT-PCR using GAPDH and β-actin as housekeeping genes to normalize gene expression. The y-axis represents the relative expression normalized to housekeeping genes (HKGs); the y-axis scale is in LOG_10_ except for αSMA. ^**^p < 0.01; ^***^p < 0.001.

Furthermore, we evaluated the expression level of genes coding for proteins of the ECM. αSMA mRNA expression was upregulated in HS (2.3-fold decrease, p < 0.01) compared to normal skin, whereas its upregulation was limited and not significant in KS, most likely because of the heterogeneous distribution between the samples (Fig. 3). As expected, overexpression of fibronectin, col1α1, col1α2 and col3α1 mRNA was observed in both pathologic scars compared to normal skin, without a difference between HS and KS.

### OSM was able to partially overcome the effect of TGFβ1 on fibroblasts *in vitro*

**Figure 4:**
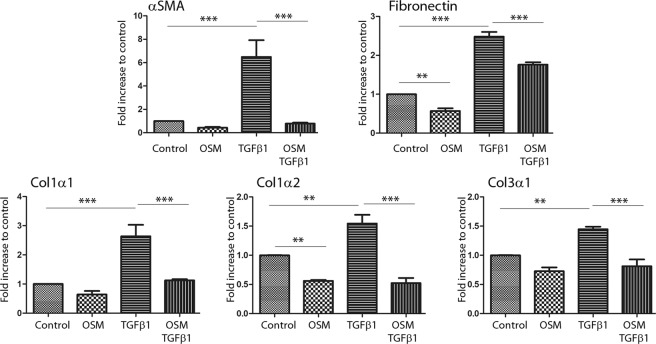
OSM counteracts the effect of TGFβ1 on ECM protein expression in normal dermal fibroblasts at the mRNA level. Dermal fibroblasts from normal skin were cultured for 24 h with or without TGFβ1 (10 ng/ml) and OSM (10 ng/ml). ECM protein gene expression was analysed by qRT-PCR using GAPDH and β-actin as housekeeping genes to normalize gene expression, which is presented as a fold increase compared to the control. The experiments were performed with fibroblasts from 4 different patients (n = 4). ^**^p < 0.01; ^***^p < 0.001.

To determine the effect of OSM on ECM protein expression in fibroblasts, normal, hypertrophic and keloid dermal fibroblasts were cultured with OSM and/or TGFβ1. As expected, TGFβ1 stimulated the mRNA expression levels of αSMA (6.4-fold increase, p < 0.001), fibronectin (2.5-fold increase, p < 0.001), col1α1 (2.4-fold increase, p < 0.001), col1α2 (1.5-fold increase, p < 0.01) and col3α1 (1.3-fold increase, p < 0.01) in normal fibroblasts. OSM alone had a discrete effect and significantly decreased (p < 0.01) fibronectin and col1α2 gene expression levels. However, when the cells were treated with both factors, OSM partially or totally suppressed the TGFβ1-induced upregulation of αSMA (8.5-fold decrease, p < 0.001), fibronectin (1.4-fold decrease, p < 0.001), col1α1 (2.3-fold decrease, p < 0.001), col1α2 (4-fold decrease, p < 0.001) and col3α1 expression (2.6-fold decrease, p < 0.001) (Fig. 4). Similar results were observed in fibroblasts isolated from hypertrophic or keloid scars (Figs S1 and S2).

**Figure 5:**
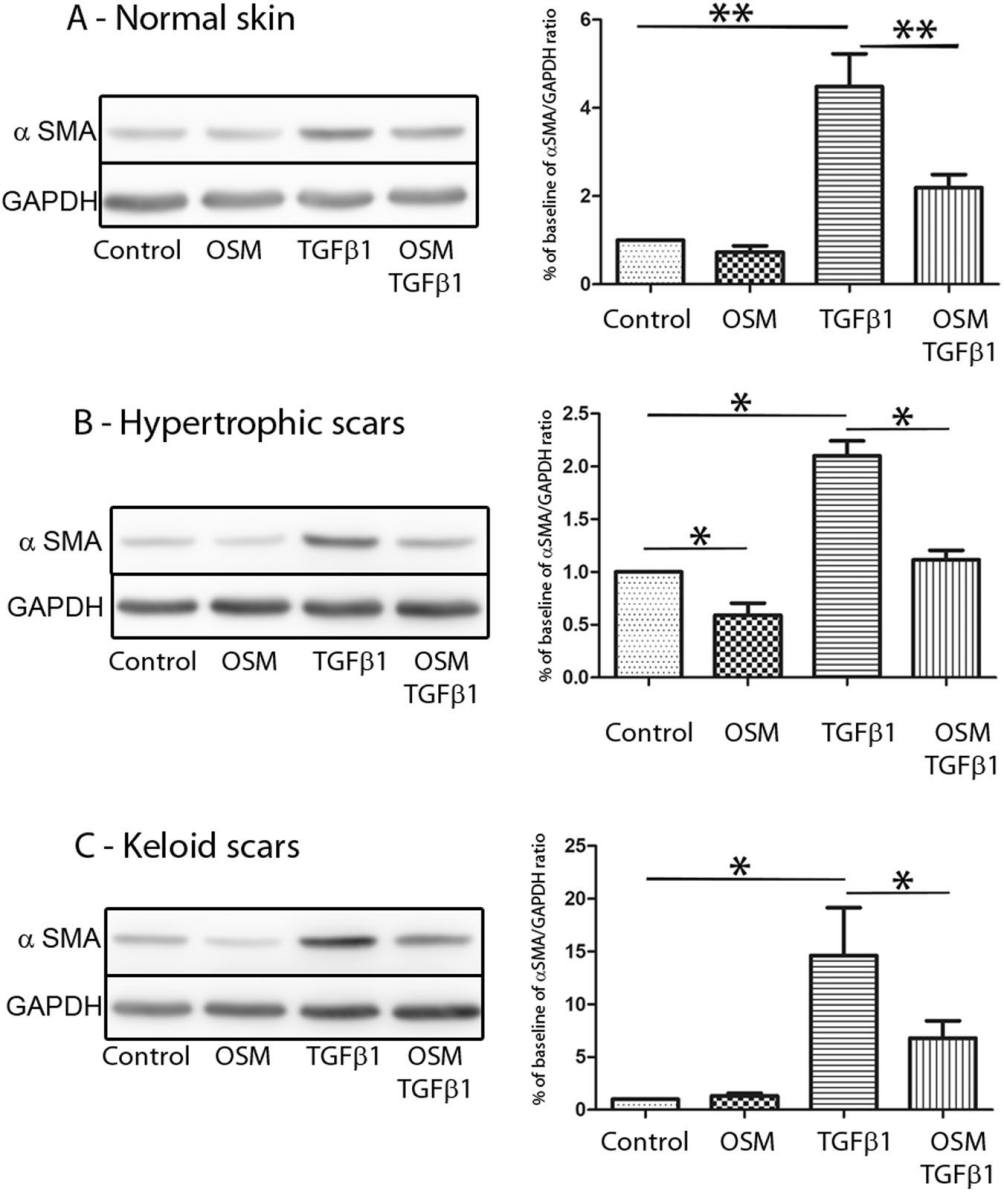
OSM counteracts the effect of TGFβ1 on ECM protein expression in dermal fibroblasts at the protein level. Dermal fibroblasts from normal skin (**A**), HS (**B**) or KS (**C**) were cultured for 48 h with or without TGFβ1 (10 ng/ml) and OSM (10 ng/ml). The cells were lysed, and proteins were analysed by western blotting. αSMA and GAPDH were immunodetected by co-incubation of specific antibodies on the same membrane. For chemiluminescence image acquisition (Fujifilm LAS-3000 imaging system) (left panel) and quantification (Fujifilm Multi GaugeV3.0 software) (right panel) of GAPDH and αSMA signals, the membranes were exposed for 10 sec and 30 sec, respectively. The experiments were performed in duplicate with fibroblasts from 4 different patients in each group (normal fibroblasts, hypertrophic fibroblasts and keloid fibroblasts, n = 4). ^*^p < 0.05; ^**^p < 0.01. The left panel is representative of one out of four independent experiments. Uncropped full-length blots are presented in the Supplementary Information (Figs S3 and S4).

αSMA protein expression under TGFβ1 and/or OSM stimulation of normal, hypertrophic and keloid dermal fibroblasts was further examined by western blot analysis. Similar results were observed at the protein level. OSM alone had a weak effect on αSMA expression. TGFβ1 increased αSMA expression levels in all types of dermal fibroblasts (p < 0.01 for normal dermal fibroblasts and p < 0.05 for hypertrophic and keloid dermal fibroblasts, Fig. 5). As observed at the transcriptional level, OSM partially reversed the TGFβ1-induced upregulation of αSMA expression in all types of dermal fibroblasts (p < 0.01 for normal dermal fibroblasts and p < 0.05 for hypertrophic and keloid dermal fibroblasts, Fig. 5).

## Discussion

Numerous studies have described the histopathologic and molecular characteristics of hypertrophic and keloid scars with specific features and occasionally controversial results^25^. Nonetheless, it is difficult to distinguish them by only using histology^26,27^ or electron microscopy^28^, especially at the beginning of their evolution^29,30^. As expected, we observed that both hypertrophic and keloid scars contain collagen bundles in the papillary dermis. These collagen bundles are aligned parallel to the epithelial surface and are thicker in the mid and reticular dermis. No clear difference in collagen fibre architecture between the two types of scars has been observed, but the presence of abundant hyalinized collagen fibres observed in keloid scars is specific^30–33^. In hypertrophic scars, the presence of myofibroblasts, characterized by αSMA protein expression, is more frequent than in keloid scars^29,32,34^. As previously described, we observed moderate acanthosis in both types of scars^34–37^. However, hypertrophic and, to a better extent, keloid scars had more Ki67-positive cells in the basal layer of the epidermis than healthy skin. We suggest that keratinocyte turnover is more active in keloid scars, while other studies have hypothesized that increased epidermal thickness is attributed to the abnormal differentiation of keratinocytes^37^. Overall, these phenotypes are in accordance with the previously reported active status of keratinocytes in the pathogenesis of hypertrophic and keloid scars^6,37–40^.

Hypertrophic and keloid scars are characterized by the excessive deposition of ECM components^41,42^. After injury, a physiological process of repair is initiated. Fibroblasts become activated and proliferate, then migrate into the wound and synthetize a scaffold of reparative tissues composed of matrix proteins, such as collagen, proteoglycan and fibronectin. The fibroblasts differentiate into myofibroblasts that express high amounts of αSMA and contribute to the initiation of wound remodelling. To date, several studies have suggested that a prolonged inflammatory phase may contribute to increased fibroblast activity and excessive scarring^6,43^. Hypertrophic and keloid scars are indeed associated with a chronic inflammation state, increased infiltration of immune cells and production of cytokines. Both types of scars overexpressed the antimicrobial peptides S100A7 and/or BD2, indicating the inflammatory status. TGFβ1, a key inductor of dermal matrix production, was overexpressed at similar levels in both types of pathologic scars compared to healthy skin, in accordance with previous studies^44–46^. Downstream of TGFβ1, the pro-fibrotic markers col1α1, col1α2, col3α1 and fibronectin were overexpressed at the same level in both types of scars.

We analysed the expression of cytokines involved in ECM metabolism and fibroblast functions. IL-1α, IL-1β and TNFα mRNA expression levels decreased in pathologic scars compared to healthy skin, especially IL-1β in keloid scars. Variations in cytokine expression have been reported with contradictory results. Using immunohistochemistry, Niessen *et al*. reported a decrease in IL-1α expression in the epidermis of hypertrophic scars, but not for IL-1β and TNFα^7^. Using a cytokine antibody array, Zhan *et al*. reported overexpression of TNFα and IL-1β in keloid scars, but not for IL-1α^11^. They also showed overexpression of IL-6 and IL-17A in keloid scars, while mRNA expression of these cytokines was not observed in our study. We also observed downregulated expression of Cox2 in both types of scars, in accordance with other studies^47^. This was consistent with the downregulated expression of IL-1β and TNFα observed in both scars since these inflammatory mediators activate Cox2 expression in dermal fibroblasts^48,49^. Overall, the inflammatory state of the scars was observed to be dependent on the age and the state of evolution of the pathology. The heterogeneity of the biopsies used in the different studies might explain the contradictory results reported in the literature.

Amongst the panel of cytokines that were analysed, OSM was the only cytokine that was increased in HS but not in KS. Regarding KS, this result is in accordance with Zhang *et al*^11^., whereas Canady *et al*. reported OSM overexpression in KS compared to healthy skin^50^.

OSM is a proinflammatory cytokine that plays a crucial role in the pathogenesis of various fibrotic diseases^15,51,52^. OSM has been reported to be a potent mediator of lung inflammation and ECM accumulation;^51^ however, the involvement of OSM in inflammation and fibrosis is unclear depending on the pathologies and the cellular microenvironment. For example, mice with pancreas-targeted OSM overexpression have been shown to develop severe localized fibrotic lesions^53^, and in dermal fibroblasts, OSM has been shown to activate the col1α2 promotor and potently induce collagen and glycosaminoglycan production^54^. Furthermore, OSM has been reported to attenuate the inflammatory response by promoting the reestablishment of homeostasis in cooperation with proinflammatory cytokines and acute phase molecules^55,56^. OSM can also inhibit TGFβ1-induced extracellular matrix protein expression in human proximal tubule cells^57,58^.

In this context, and since OSM was overexpressed in HS but not in KS, we speculated whether OSM could protect against a chronic fibrosis mechanism that occurs in keloid scars. We hypothesize that overexpression of OSM in hypertrophic scars could contribute to the regression of inflammation and normal scaring. OSM can be involved in the wound healing process by multiple mechanisms. OSM induces acanthosis, migration and an inflammatory state of keratinocytes^20^ and promotes dermal fibroblast proliferation, ECM synthesis and angiogenesis^54,59,60^. In this study, OSM alone had a discrete inhibitory effect on fibronectin and col1α2 gene expression in dermal fibroblasts but strongly counteracted the inducing effect of TGFβ1 on αSMA, fibronectin, col1α1, col1α2, and col3α1 gene expression. The OSM effect on these fibrosis markers was similar between normal, hypertrophic and keloid fibroblasts. Therefore, we suggest that the contribution of OSM to scar regression is attributed to OSM-enhanced expression rather than fibroblast sensitivity to this cytokine.

Overall, we reported a specific OSM overexpression in HS but not in KS, which suggested that OSM could protect against excessive scarring by inhibiting TGFβ1-induced ECM protein expression in fibroblasts, as supported by the *in vitro* experiments. In the absence of highly effective treatments for keloid scars, the use of OSM may offer promising strategies for the development of new therapeutic treatments.

## Patients, Materials and Methods

### Prospective clinical study

This study included 18 adult patients presenting hypertrophic (n = 9) or keloid (n = 9) scars. All of our studies involving human tissues were approved by the Institutional Ethics Committee on Human Experimentation (Comité de Protection des Personnes Ouest III) of the Poitou-Charentes Region. This study was conducted according to the Declaration of Helsinki principles, and oral informed consent was obtained from participants before inclusion. Skin biopsies were obtained during the surgical treatment of the scars. Skin biopsies of control subjects were obtained from surgical samples of healthy abdominal or breast skin. The biopsies were immediately frozen in liquid nitrogen before RNA extraction, stored in formalin for histology and immunohistochemistry, or immediately treated for fibroblast extraction.

### Histology and immunohistochemistry on human skin

Histology and immunohistochemistry were performed on tissue sections from formalin-fixed paraffin-embedded tissue blocks of patient skin. Four-micrometre-thick skin sections were stained with haematoxylin and eosin (H&E) and used for routine diagnosis of the scars. For immunohistochemistry, 4 µm serial sections were cut from a tissue block, deparaffinized in xylene and hydrated in a graded series of alcohol. After antigen retrieval with cell conditioning solution (CC1 – Ventana Medical Systems, Tucson, AZ, USA), staining was performed using a BenchMark automated staining system (Ventana Medical Systems) for Ki67 (IgG1, clone MIB-1, 1:100 dilution, DakoCytomation, Glostrup, Denmark) or α smooth muscle actin (**α**SMA) (IgG2a, clone 1A4, 1:800 dilution, DakoCytomation). An ultraView universal DAB detection kit (Ventana Medical Systems) was used, and slides were counterstained with haematoxylin. Appropriate irrelevant polyclonal or monoclonal antibodies were used as negative controls. Basal keratinocytes expressing Ki67 were counted in three representative areas for each patient, and epidermal thickness was measured using cellSens software (Olympus Corporation, Tokyo, Japan). We performed a quantitative analysis by scoring the immune cell infiltrate and αSMA expression.

### Quantitative RT-PCR Analysis

Total RNA from skin biopsies (including epidermis and dermis) and fibroblasts was isolated using a NucleoSpin® RNA II kit (Macherey-Nagel, Hoerdt, France) and reverse-transcribed with SuperScript® II reverse transcriptase (Invitrogen, Life Technologies, Carlsbad, CA, USA) according to the manufacturer’s instructions. Quantitative real-time PCR was conducted using a Light Cycler-FastStart DNA MasterPlus SYBR® Green I kit and a LightCycler 480 system (Roche Diagnostics, Meylan, France). The reaction components consisted of 1x DNA Master Mix and 0.5 µM HPLC-purified sense and anti-sense oligonucleotides purchased from Eurogentec (Eurogentec France, Angers, France) and designed using Primer3 software. Relative RNA expression was determined according to the ∆CT method (relative expression = 2exp(∆CT) = 2exp(CT target – CT housekeeping)). For normalizing the expression levels, we used the mean CT of 2 housekeeping genes (glyceraldehyde-3-phosphate dehydrogenase and β-actin). The graphs show the relative expression of each gene compared to the expression of the housekeeping genes or the fold increase compared to the control (the relative expression of stimulated cells compared to the relative expression of control cells).

### Cell extraction and culture

Dermal fibroblasts were obtained from surgical samples of healthy breast skin or from hypertrophic or keloid scars. Dermal tissues were cut into 1–2 mm^3^ pieces and incubated in a culture plate with Dulbecco’s-modified Eagle’s medium (DMEM) supplemented with 2 mM L-glutamine, 10% foetal calf serum (FCS), 100 u/ml penicillin and 100 µg/ml streptomycin (all from Thermo Fisher Scientific, Waltham, MA, USA) until the fibroblasts migrated out from the pieces of tissue. For all experiments, cells were used at the 3^rd^ passage.

For qRT-PCR and western blot analyses, cells were starved for 24 h in DMEM supplemented with 0.5% FCS before being treated with or without 10 ng/ml recombinant human TGFβ1 (PeproTech, Rocky Hill, NJ, USA) and human OSM (R&D Systems Europe, Lille, France) alone or in combination for 24 h for mRNA quantification or 48 h for western blot.

### Western blotting

After 48 h of stimulation, fibroblast lysis was performed as previously described^61^. After separation on a 12% SDS-PAGE gel, proteins were transferred to nitrocellulose membranes (GE Healthcare, Chicago, IL, USA) by electroblotting. Immunodetection of αSMA and GAPDH was performed by co-incubation with rabbit anti-αSMA Ab (Novus Biologicals, Centennial, CO, USA) and mouse anti-GAPDH mAb (Novus, clone 2D4A7), followed by co-incubation with anti-rabbit and anti-mouse IgG peroxidase-conjugated polyclonal Abs (Sigma-Aldrich, St. Louis, MO, USA). Peroxidase activity was detected by chemiluminescence (Luminata HRP substrate from Merck Millipore Burlington, MA, USA) and analysed using an LAS-3000 imaging system (Fujifilm, Tokyo, Japan) followed by quantification using Multi Gauge V3.0 software (Fujifilm). Each membrane was exposed for 10 sec and 30 sec for GAPDH and αSMA quantification, respectively. The ratios of αSMA/GAPDH were calculated and are shown in the corresponding figures. The experiment was repeated four times, and the images are representative of one experiment.

### Statistical analysis

For the *ex vivo* experiments, statistical analysis was performed using Kruskal-Wallis one-way ANOVA with Dunn’s post-test.

For the *in vitro* experiments, statistical analysis was performed using the Mann-Whitney U test. p-values less than 0.05 were considered statistically significant. The data are presented as the mean and SEM.

## Supplementary information

Supplementary Data

## Data Availability

All data generated or analysed during this study are included in this published article (and the Supplementary Information files).
