# Supplementary material for: Oncostatin M exerts a protective effect against excessive scarring by counteracting the inductive effect of TGFβ1 on fibrosis markers
Source: Sci Rep. 2019 Feb 14;9:2113. doi: 10.1038/s41598-019-38572-0 (PMC6376164; doi:10.1038/s41598-019-38572-0)
Supplement: Supplementary file 1 — Supplementary Data [file 41598_2019_38572_MOESM1_ESM.pdf]

Oncostatin M exerts a protective effect against excessive scarring by counteracting the inductive effect of TGFβ1 on fibrosis markers

Vincent Huguier, Jean-Philippe Giot, Marie Simonneau, Pierre Levillain, Sandrine Charreau, Martine Garcia, Jean-François Jégou, Charles Bodet, Franck Morel, Jean-Claude Lecron, Laure Favot.

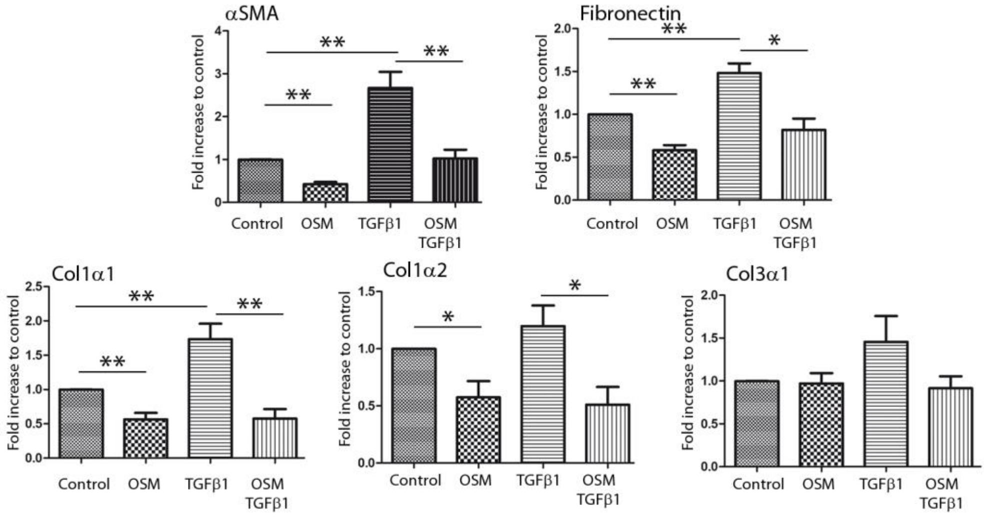

Figure S1

Figure S1 : OSM counteracts the effect of TGFβ1 on ECM proteins expression in dermal fibroblasts from hypertrophic scars at the mRNA level.

Dermal fibroblasts from hypertrophic scars were cultured for 24h with or without TGFβ1 (10 ng/ml) and OSM (10 ng/ml). ECM proteins gene expression was quantified by qRT-PCR using GAPDH and βactin as housekeeping genes to normalize gene expression and expressed as fold increase to control. Experiments have been performed with fibroblasts from 4 different patients n=4, *$p<0.05$; ** $p<0.01$

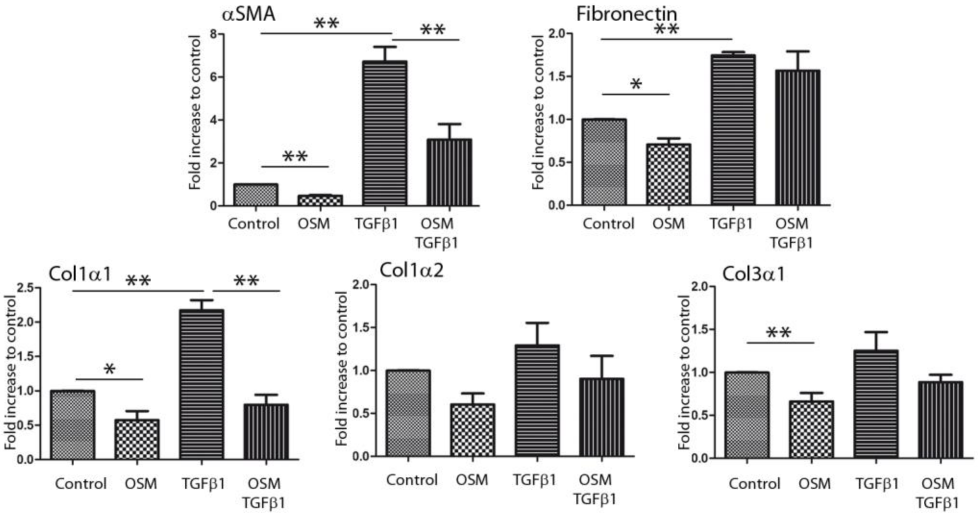

Figure S2

Figure S2 : OSM counteracts the effect of TGFβ1 on ECM proteins expression in dermal fibroblasts from keloid scars at the mRNA level.

Dermal fibroblasts from keloid scars were cultured for 24h with or without TGFβ1 (10 ng/ml) and OSM (10 ng/ml). ECM proteins gene expression was quantified by qRT-PCR using GAPDH and βactin as housekeeping genes to normalize gene expression and expressed as fold increase to control. Experiments have been performed with fibroblasts from 4 different patients n=4, *p<0.05; ** p<0.01

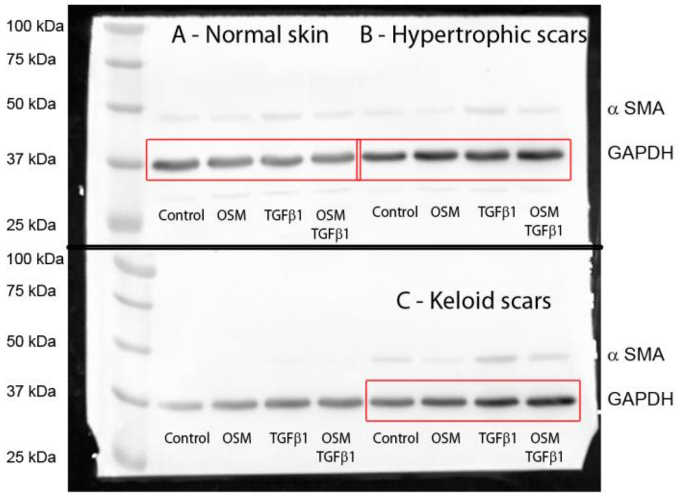

Figure S3

Figure S3. Uncropped images relative to Figure 5.

Red outlines represent GAPDH immune-detection shown in Figure 5 (left panel) after 10 sec chemiluminescence image acquisition (Fujifilm LAS-3000 imaging system) and used for quantification (Fujifilm Multi GaugeV3.0 software).

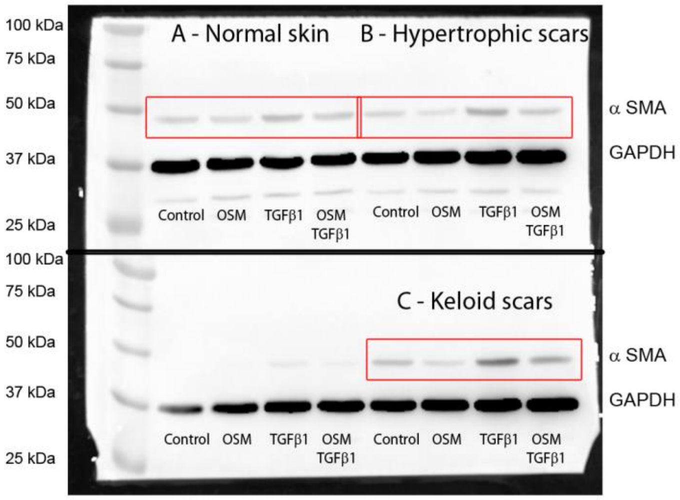

Figure S4

Figure S4. Uncropped images relative to Figure 5.

Red outlines represent α SMA immune-detection shown in Figure 5 (left panel) after 30 sec chemiluminescence image acquisition (Fujifilm LAS-3000 imaging system) and used for quantification (Fujifilm Multi GaugeV3.0 software).
